# Prevalence and clinical significance of antiphospholipid antibodies in patients with coronavirus disease 2019 admitted to intensive care units: a prospective observational study

**DOI:** 10.1007/s00296-021-04875-7

**Published:** 2021-05-05

**Authors:** Mostafa Najim, Alaa Rahhal, Fadi Khir, Amer Hussien Aljundi, Safae Abu Yousef, Feryal Ibrahim, Aliaa Amer, Ahmed S. Mohamed, Samira Saleh, Dekra Alfaridi, Ahmed Mahfouz, Sumaya Alyafei, Faraj Howady, Mohamad Khatib, Samar A. Alemadi

**Affiliations:** 1 Internal Medicine Department, Hamad Medical Corporation, Doha, Qatar; 2 Pharmacy Department, Hamad Medical Corporation, Doha, Qatar; 3 Laboratory Department, Hamad Medical Corporation, Doha, Qatar; 4 Nursing Department, Hamad Medical Corporation, Doha, Qatar; 5 Infectious Diseases Department, Hamad Medical Corporation, Doha, Qatar; 6 Medical Intensive Care Department, Hamad Medical Corporation, Doha, Qatar; 7 Rheumatology Department, Hamad Medical Corporation, Doha, Qatar

**Keywords:** COVID-19, Antiphospholipid antibodies, Arterial thrombosis, Venous thrombosis, Coagulopathy, Critically ill patients

## Abstract

Coronavirus disease 2019 (COVID-19) increases the risk of coagulopathy. Although the presence of antiphospholipid antibodies (aPLs) has been proposed as a possible mechanism of COVID-19-induced coagulopathy, its clinical significance remains uncertain. Therefore, this study aimed to evaluate the prevalence and clinical significance of aPLs among critically ill patients with COVID-19. This prospective observational study included 60 patients with COVID-19 admitted to intensive care units (ICU). The study outcomes included prevalence of aPLs, and a primary composite outcome of all-cause mortality and arterial or venous thrombosis between antiphospholipid-positive and antiphospholipid-negative patients during their ICU stay. Multiple logistic regression was used to assess the influence of aPLs on the primary composite outcome of mortality and thrombosis. A total of 60 critically ill patients were enrolled. Among them, 57 (95%) were men, with a mean age of 52.8 ± 12.2 years, and the majority were from Asia (68%). Twenty-two patients (37%) were found be antiphospholipid-positive; 21 of them were positive for lupus anticoagulant, whereas one patient was positive for anti-β2-glycoprotein IgG/IgM. The composite outcome of mortality and thrombosis during their ICU stay did not differ between antiphospholipid-positive and antiphospholipid-negative patients (4 [18%] vs. 6 [16%], adjusted odds ratio 0.98, 95% confidence interval 0.1–6.7; *p* value = 0.986). The presence of aPLs does not seem to affect the outcomes of critically ill patients with COVID-19 in terms of all-cause mortality and thrombosis. Therefore, clinicians may not screen critically ill patients with COVID-19 for aPLs unless deemed clinically appropriate.

## Introduction

The novel coronavirus infection (also known as coronavirus disease 2019 [COVID-19]) significantly contributes to the increased mortality in many countries, with a continuously increasing number of infected cases worldwide [1]. One of the poor prognostic features in critically ill patients with COVID-19 is the development of coagulopathy [2]. Patients who develop sepsis due to COVID-19 are at risk of developing coagulopathy, a condition associated with poor outcomes, as demonstrated by a retrospective analysis conducted in China [3]. The development of disseminated intravascular coagulation (DIC) on day 4 was observed in 71.4% of patients who died compared to 0.6% of patients who survived. Moreover, a significantly increased D-dimer level and prothrombin time (PT) and decreased fibrinogen levels in non-survivors were also observed. Consequently, the International Society on Thrombosis and Homeostasis recently recommended that all hospitalized patients with COVID-19 receive a prophylactic-dose of low-molecular-weight heparin (LMWH) unless they have contraindications defined as active bleeding and platelet count of < 25 × 10^9^/L [4].

Antiphospholipid syndrome (APS) is a prothrombotic state characterized by arterial or venous thrombosis in the setting of persistent laboratory evidence of antiphospholipid antibodies (aPLs) [5]. According to the 2018 Scientific Standardisation Subcommittee of the International Society on Thrombosis and Haemostasis on APS classification criteria, laboratory criteria include screening for lupus anticoagulant, IgM and/or IgG anticardiolipin, and IgM and/or IgG anti-β2-glycoprotein antibodies [6]. The mechanism of COVID-19-induced coagulopathy is not yet well established; however, a case series in China in the early period of the pandemic reported that three ICU patients with COVID-19 had positive aPLs, including anticardiolipin IgA and anti-β2-glycoprotein IgA and IgG, and all of them had multiple cerebral infarcts [7]. Additionally, a case series of two patients with COVID-19 who developed significant thrombotic events during their hospital stay reported that the hypercoagulopathy workup revealed positive IgM and IgG anticardiolipin [8]. Nevertheless, the prevalence of aPLs in critically ill patients with COVID-19 varies in the literature [9–11].

In fact, viral infections, in particular, have been associated with transient aPLs, such as hepatitis C and human immunodeficiency virus [5, 12, 13]. Despite the association between viral infections and aPLs, its clinical impact on thrombotic events has not yet been well defined [14]. Given the conflicting prevalence of aPLs among critically ill patients with COVID-19 and the lack of a robust association between COVID-19-induced aPLs and clinical outcomes of thrombosis and mortality, this prospective observational study aimed to evaluate the prevalence and clinical importance of aPLs among critically ill patients with COVID-19.

## Materials and methods

### Study setting

This study was conducted at the Hazm Mebaireek General Hospital (HMGH), a general tertiary hospital part of the Hamad Medical Corporation (HMC), which is a dominant healthcare provider in Qatar. By the end of March 2020, HMGH was designated as the first COVID-19 treatment facility in Qatar to enable HMC to provide high-quality care for patients with COVID-19. The hospital has 12 intensive care units (ICU) with a maximum bed capacity of 236 for patients with COVID-19.

### Study design and oversight

This prospective observational cohort study was conducted to evaluate the prevalence and clinical significance of aPLs, including anticardiolipin IgG/IgM, anti-β2-glycoprotein IgG/IgM, and lupus anticoagulant among COVID-19 patients admitted to the ICUs in HMGH. The study was approved by the HMC medical research center and institutional review board (MRC-05-038) on June 21, 2020. Written informed consent was obtained from conscious and alert patients under strict measures to minimize transmission of infection while obtaining consent based on local infection control policy. For the unconscious intubated patients, the patient’s next kin or legal guardian was called and consented over the phone through an approved “telephone script” by the HMC medical research center and institutional review board.

### Patients and eligibility criteria

Eligible patients were adults aged > 18 years who were admitted to HMGH ICUs with a diagnosis of COVID-19 confirmed via a positive polymerase chain reaction (PCR) test [15].

Patients were excluded if they had a known history of thrombophilia, including APS, had a history or active autoimmune and autoinflammatory rheumatic diseases, had a confirmed thrombotic event immediately before enrollment, were receiving therapeutic anticoagulation before COVID-19 diagnosis, had active malignancy, were pregnant, or refused to sign the consent.

### Study procedures

This prospective observational cohort study consisted of three main phases: (1) estimating the prevalence of aPLs, including anticardiolipin IgG/IgM, anti-β2-glycoprotein IgG/IgM, and lupus anticoagulant among critically ill patients with COVID-19 by screening for aPLs within 72 h of ICU admission; (2) assessing the clinical significance of aPLs among critically ill patients with COVID-19 by determining the incidence of arterial and venous thrombotic events and mortality during their ICU stay; and (3) estimating the prevalence of aPLs among patients with COVID-19 during the development of thrombosis.

Upon admission to the ICU, patients with COVID-19 who met the study’s eligibility criteria were consecutively screened for aPLs (anticardiolipin IgG/IgM, anti-β2-glycoprotein IgG/IgM, and lupus anticoagulant) after obtaining informed consent. Then, recruited patients were followed during their ICU stay for the development of the study’s clinical outcomes, such as arterial or venous thrombosis and mortality, which were then compared among those who were found to be positive for aPLs and those without the antibodies at baseline screening. A repeat screening for all antibodies (anticardiolipin IgG/IgM, anti-β2-glycoprotein IgG/IgM, and lupus anticoagulant) was performed for patients who developed a thrombotic event during the same ICU admission. Patients were followed up for 60 days or until death or discharge from the ICU, whichever came first.

Enrolled participants were stratified into two main groups based on their aPL initial screening results: (1) first group: critically ill patients with COVID-19 and at least one positive antiphospholipid assay (antiphospholipid-positive group); (2) second group: critically ill patients with COVID-19 and negative aPLs (antiphospholipid-negative group).

Data were collected from the HMC electronic medical records system using Cerner^®^. Relevant data were manually extracted using a pretested data collection form.

### Outcomes

The outcomes measured in this study were as follows: (1) prevalence of aPLs among critically ill patients with COVID-19; (2) a primary composite outcome of mortality and arterial or venous thrombosis among antiphospholipid-positive and antiphospholipid-negative patients during their ICU admission; (3) secondary outcomes, including all-cause mortality, venous thrombosis, arterial thrombosis, ICU discharge, time to mortality, and time to ICU discharge among antiphospholipid-positive and antiphospholipid-negative patients during their ICU admission; and (4) prevalence of aPLs among patients with COVID-19 during the development of thrombosis.

Antiphospholipid screening was performed according to standardized procedures recommended by the International Society on Thrombosis and Haemostasis [6, 16–18]. For lupus anticoagulant testing, three-step testing using the Siemens dRVVT Screen and Siemens dRVVT Confirm assays were used, as recommended by the Scientific Standardisation Subcommittee on Lupus Anticoagulant/Phospholipid Antibodies [17]. Anticardiolipin IgG/IgM and anti-β2-glycoprotein IgG/IgM were performed according to the ISTH guidelines [18]. Anticardiolipin IgG/IgM and anti-β2-glycoprotein IgG/IgM were measured using a fluoro enzyme immunoassay (FEIA) by Phadia 250 platform. Anticardiolipin IgG/IgM was considered positive at a cutoff value of > 40 GPL or MPL, whereas anti-β2-glycoprotein IgG/IgM was deemed to be positive at a cutoff value of > 10 U/mL based on the based on the manufacturer’s recommendations.

### Statistical analysis

For the sample size determination, given the disease novelty, local data on the prevalence of thrombosis or APS among patients with COVID-19 in Qatar could not be utilized upon designing the study in April 2020. With the assumption that the predicted prevalence of antiphospholipid among patients with COVID-19 is 91%, as reported by Bowles L et al*.* in May 2020 in the United Kingdom [9], a 95% confidence interval (CI) with a *Z *value of 1.96, and a precision value of 5%, the minimum determined effective sample size based on these assumptions was 60 patients.

Statistical analyses were performed using the Statistical Package for Social Sciences program version 24.0 (IBM SPSS Statistics for Windows; IBM Corp, Armonk, NY). The prevalence of aPLs was reported as frequencies and percentages. Descriptive statistics in the form of mean and standard deviations or median and interquartile ranges were calculated for interval variables and percentages for categorical variables. Chi-square or Fisher’s exact tests were used for categorical variables to compare between the groups. Student’s *t* test was used for normally distributed variables and Mann–Whitney *U* test for non-normally distributed interval variables between the study’s groups. Multiple logistic regression adjusted for pre-specified clinically relevant covariates, including the use of venous thromboembolism (VTE) prophylaxis before ICU admission, switching VTE prophylaxis to therapeutic anticoagulation following the local protocol, and ICU admission due to acute respiratory distress syndrome (ARDS) was used to assess the influence of the presence of aPLs on the primary composite outcome of mortality and thrombosis. An additional analysis was conducted to confirm the results of the primary analysis. In the confirmatory multiple logistic regression, all baseline imbalances with *p* values of < 0.05 were included in the multiple logistic regression model. All *p *values were two-sided, and results with *p* values of < 0.05 were used to indicate statistical significance.

## Results

### Patient selection and baseline characteristics

From June 26 to August 5, 2020, we identified a total of 117 patients with confirmed COVID-19 infection upon ICU admission; 60 of them were found to be eligible for study enrollment. After screening for aPLs upon admission to the ICU, 22 participants were found to have positive aPLs either single or in combination and hence included in the antiphospholipid-positive arm, whereas the remaining 38 participants had a negative screening for aPLs and, therefore, included in the antiphospholipid-negative arm.

The baseline demographic, laboratory, and clinical characteristics of the study population (*n* = 60) are shown in Table 1. with characteristics comparison between critically ill patients with COVID-19 with positive aPLs (*n* = 22) and those with negative aPLs (*n* = 38). Among the 60 patients enrolled, 57 (95%) were men, with a mean age of 52.8 ± 12.2 years. The majority of them were from Asia (68%), followed by the Middle East (30%). Additionally, half of the study population had a past medical history of hypertension, whereas 42% had diabetes mellitus.

**Table 1:** Baseline demographic, laboratory, and clinical characteristics of COVID-19 patients (*n* = 60)

Characteristic	All patients (*n* = 60), *n* (%)	aPL(s) positive (*n* = 22), *n* (%)		aPL(s) negative (*n* = 38), *n* (%)	*p *value
Male gender	57 (95)	21 (96)		36 (95)	1^*^
Age [years]^a^	52.8 ± 12.2	49.4 ± 11.5		54.8 ± 12.3	0.097
Geographical region of origin					0.450
Asia	41 (68)	17 (77)		24 (63)	
Middle East	18 (30)	5 (23)		13 (34)	
Africa	1 (2)	0		1 (3)	
Smoking^b^					0.067^*^
Never	32 (53)	15 (100)		17 (77)	
Ex-smoker	5 (8)	0		5 (23)	
Medical history					
Hypertension	30 (50)	9 (41)		21 (55)	0.284
Diabetes	25 (42)	8 (36)		17 (45)	0.526
Chronic kidney disease	4 (7)	1 (5)		3 (8)	1^*^
Coronary artery disease	5 (8)	2 (9)		3 (8)	1^*^
Ischemic stroke	3 (5)	0		3 (8)	0.292^*^
Venous thromboembolism	1 (2)	1 (5)		0	0.367^*^
Symptoms at COVID-19 diagnosis					
Fever	47 (78)	16 (73)	31 (82)		0.520^*^
Shortness of breath	45 (75)	19 (86)	26 (68)		0.122
Vomiting	4 (7)	1 (5)	3 (8)		1^*^
Diarrhea	7 (12)	4 (18)	3 (8)		0.405^*^
Myalgia	13 (22)	6 (27)	7 (18)		0.520^*^
Fatigue	10 (17)	6 (27)	4 (11)		0.149^*^
Headache	6 (10)	3 (14)	3 (8)		0.659^*^
Productive cough	2 (3)	1 (5)	1 (3)		1^*^
Non-productive Cough	47 (48)	19 (86)	28 (74)		0.338^*^
Laboratory findings before ICU admission					
Serum creatinine [μmol/L]^c^	102 (41)	105 (33)	98 (49)		0.404^**^
Alanine aminotransferase [U/L]^c^	32 (33)	35 (34)	29 (29)		0.243^**^
Aspartate transaminase [U/L]^c^	42 (34)	46 (35)	40 (26)		0.178^**^
Platelet count [× 10^3^/μL]^c^	202 (79)	205 (72)	198 (79)		0.693^**^
Hemoglobin [g/dL]^c^	13.9 ± 2.2	13.9 ± 1.7	13.9 ± 2.4		0.910
Ferritin [μg/L]^c^	625 (940)	682 (1744)	539 (939)		0.400^**^
D-Dimer [mg/L]^c^	0.68 (0.71)	0.55 (0.86)	0.72 (0.66)		0.522^**^
Prothrombin time [second]^a^	12.6 ± 1.2	12.0 ± 1.3	12.8 ± 1.2		0.131
Activated partial thromboplastin time [second]^a^	31.5 ± 4.3	33.1 ± 4.3	31.1 ± 4.3		0.281
Fibrinogen [g/L]^a^	5.9 ± 1.9	5.8 ± 1.8	5.9 ± 2.0		0.952
C-reactive protein [mg/L]^a^	81 (88)	106 (131)	77 (70)		0.144^**^
Procalcitonin [ng/mL]^c^	0.21 (0.71)	0.12 (25.31)	0.28 (0.68)		0.741^**^
Interleukin-6 [pg/mL]^c^	36 (60)	44 (301)	34 (60)		0.727^**^
COVID-19 treatment before ICU admission					
Azithromycin	35 (58)	11 (50)	24 (63)		0.319
Hydroxychloroquine	3 (5)	2 (9)	1 (3)		0.548^*^
Oseltamivir	3 (5)	2 (9)	1 (3)		0.584^*^
Cephalosporin					0.227
None	18 (30)	10 (45)	8 (21)		
Ceftriaxone	23 (38)	7 (32)	16 (42)		
Cefuroxime	18 (30)	5 (23)	13 (34)		
Others	1 (2)	0	1 (3)		
Antipseudomonal antibiotic	18 (30)	6 (27)	12 (32)		0.726
Systemic corticosteroid					0.103
None	18 (30)	11 (50)	7 (18)		
Dexamethasone	38 (63)	10 (45)	28 (73)		
Hydrocortisone	2 (3)	1 (5)	1 (3)		
Prednisolone	1 (2)	0	1 (3)		
Methylprednisolone	1 (2)	0	1 (3)		
Favipiravir	32 (53)	9 (41)	23 (61)		0.142
Convalescent plasma	5 (8)	2 (9)	3 (8)		1^*^
Tocilizumab	3 (5)	0	3 (8)		0.292^*^
VTE prophylaxis before ICU admission	46 (77)	10 (48)	36 (95)		< 0.001^*^
Admitted from medical unit to ICU	37 (62)	7 (32)	30 (80)		< 0.001
Reason for ICU admission					0.351
Desaturation	32 (53)	11 (50)	21 (55)		
Acute respiratory distress syndrome	22 (37)	10 (45)	12 (32)		
Diabetic ketoacidosis	4 (7)	0	4 (10)		
Hypotension requiring resuscitation	2 (3)	1 (5)	1 (3)		
Duration from positive COVID-19 PCR to ICU admission [days]^c^	4 (6)	3 (5)	4 (5)		0.281^**^
Laboratory findings upon ICU admission					
Serum creatinine [μmol/L]^c^	87 (36)	86 (38)	87 (37)		0.896^**^
Alanine aminotransferase [U/L]^c^	43 (34)	49 (40)	40 (33)		0.623^**^
Aspartate transaminase [U/L]^c^	45 (36)	41 (36)	46 (45)		0.718^**^
Platelet count [× 10^3^/μL]^a^	251 ± 83	251 ± 80	251 ± 86		1
Hemoglobin [g/dL]^a^	13.2 ± 2.2	13.5 ± 1.8	13.0 ± 2.5		0.448
Ferritin [μg/L]^c^	820 (908)	814 (2223)	825 (845)		0.172^**^
D-Dimer [mg/L]^c^	0.84 (1.08)	0.80 (2.14)	0.87 (1.07)		0.707^**^
Prothrombin time [second]^c^	12.8 (2.3)	13.3 (2.8)	12.6 (1.5)		0.068^**^
Activated partial thromboplastin time [second]^a^	29.7 ± 4.8	30.6 ± 5.8	29.3 ± 4.1		0.319
Fibrinogen [g/L]^a^	6.1 ± 1.6	6.8 ± 1.4	5.6 ± 1.6		0.005
C-reactive protein [mg/L]^c^	80 (110)	165 (171)	58 (54)		< 0.001^**^
Procalcitonin [ng/mL]^c^	0.23 (0.55)	0.26 (0.51)	0.19 (0.59)		0.590^**^
Interleukin-6 [pg/mL]^c^	27 (84)	32 (74)	23 (104)		0.675^**^
COVID-19 treatment during ICU Admission					
Azithromycin	13 (22)	7 (32)	6 (16)		0.197^*^
Hydroxychloroquine	0	0	0		–
Antipseudomonal antibiotic	45 (75)	16 (73)	29 (76)		0.757
MRSA-covering antibiotic	26 (43)	11 (50)	15 (40)		0.428
Systemic corticosteroid	57 (95)	21 (96)	36 (95)		1^*^
Favipiravir	47 (78)	16 (73)	31 (82)		0.520^*^
Convalescent plasma	23 (38)	10 (46)	13 (34)		0.388
Interferon β-1b	3 (5)	3 (14)	0		0.045^*^
Tocilizumab	6 (10)	3 (14)	3 (8)		0.659^*^
VTE prophylaxis	60 (100)	22 (100)	38 (100)		–
VTE prophylaxis to therapeutic according to local protocol	11 (18)	3 (14)	8 (21)		0.731^*^
ICU length of stay [days]^c^	9 (13)	9 (15)	9 (12)		0.994^**^

*aPL* antiphospholipid antibody; *ICU* intensive care unit; *PCR* polymerase chain reaction; *MRSA* Methicillin-resistant Staphylococcus aureus

^a^Values expressed as mean ± standard deviation; ^b^Data available for 61% of study population; ^c^Values expressed as median (interquartile range); ^*^p value calculated using Fisher’s exact test; ^**^p value obtained using Mann–Whitney test

As shown in Table 1, at the time of COVID-19 diagnosis, the majority of patients in both arms complained of fever (73% in the antiphospholipid-positive arm versus 82% in the antiphospholipid-negative arm, *p* value = 0.520), shortness of breath (86% vs. 68%, *p* value = 0.122), and nonproductive cough (86% vs. 74%, *p* value = 0.338). Laboratory investigations prior to ICU admission were balanced between study groups, as shown in Table 1. Similarly, treatment options for COVID-19 utilized during the hospital stay before ICU admission were balanced between the antiphospholipid-positive and antiphospholipid-negative arms, as shown in Table 1. Although fewer patients with antiphospholipid-positive arm were on VTE prophylaxis prior to ICU admission than those with antiphospholipid-negative arm (48% vs. 95%, *p* value < 0.001), significantly more patients with antiphospholipid-negative arm were transferred from a medical unit to ICU rather than admitted directly to ICU compared to those with antiphospholipid-positive arm (80% vs. 32%, *p *value < 0.001), which could explain the difference in VTE prophylaxis use between the two arms.

Desaturation was the most common reason for ICU admission in the two arms (50% vs. 55%), followed by ARDS (45% vs. 32%). Upon ICU admission, laboratory findings, including serum creatinine, liver enzymes, platelet count, hemoglobin, ferritin, D-dimer, prothrombin time, aPTT, procalcitonin, and interleukin-6, were balanced between the two arms, as shown in Table 1. However, the median C-reactive protein (CRP) and mean fibrinogen levels were higher in the antiphospholipid-positive than in the antiphospholipid-negative arm (CRP = 165 mg/L [interquartile range, 171] vs. 58 mg/L [interquartile range, 54]; *p *value < 0.001, fibrinogen = 6.8 g/L ± 1.4 vs. 5.6. ± 1.6; *p *value = 0.005), respectively. During the ICU stay, all study participants were on VTE prophylaxis, and none of them were administered hydroxychloroquine. Approximately three-quarters of patients in both arms were administered an antipseudomonal antibiotic (73% vs. 76%, *p* value = 0.757), and almost half of the patients were administered a methicillin-resistant *Staphylococcus aureus* (MRSA)-covering antibiotic (50% vs. 40%, *p *value = 0.428). Approximately all patients in both arms received systemic corticosteroids (96% vs. 95%, *p* value = 1). Although interferon β-1b was used in only 5% of the study population, its use in the antiphospholipid-positive arm was statistically more significant than those in the antiphospholipid-negative arm, as shown in Table 1 (14% vs. 0, *p* value = 0.045).

### Outcomes

#### Prevalence of antiphospholipid antibodies

The overall prevalence of aPLs, defined as detection of any aPL, including anticardiolipin IgG/IgM, anti-β2-glycoprotein IgG/IgM, or lupus anticoagulant among critically ill patients with COVID-19 within 72 h of ICU admission was 37%, as shown in Table 2. Lupus anticoagulant was the most commonly identified among the screened antibodies as it was detected in 21 (35%) of the study population, whereas only one patient had anti-β2-glycoprotein IgG/IgM.

**Table 2:** Prevalence of antiphospholipid antibodies among COVID-19 patients upon ICU admission

aPL(s)	Prevalence, *n* (%) *n* = 60
Any aPL	22 (37)
IgG anti-β2GPI	1 (2)
IgM anti-β2GPI	1 (2)
IgG aCL	0
IgM aCL	0
Lupus anticoagulant	21 (35)
LA + IgG aCL	0
LA + IgM aCL	0
LA + IgG anti-β2GPI	0
LA + IgM anti-β2GPI	1 (2)
LA + IgG aCL + IgM aCL	0
LA + IgG aCL + IgG anti-β2GPI	0
LA + IgG aCL + IgM anti-β2GPI	0
LA + IgM aCL + IgG anti-β2GPI	0
LA + IgM aCL + IgM anti-β2GPI	0
LA + IgG anti-β2GPI + IgM anti-β2GPI	0
LA + IgG aCL + IgM aCL + IgG anti-β2GPI + IgM anti-β2GPI	0

*aPL* antiphospholipid antibody, *anti-β2GPI* anti-b2 glycoprotein-I antibody, *aCL *anticardiolipin antibody, *ICU *intensive care unit, *LA *lupus anticoagulant

#### Clinical outcomes of critically ill patients with COVID-19

A total of five thrombotic events were diagnosed among study participants during their ICU stay. Remarkably, thrombotic events included two myocardial infarction events, two pulmonary embolism events, and one acute right iliac thrombosis.

The primary composite outcome of mortality or thrombosis during ICU stay adjusted for the use of VTE prophylaxis prior to ICU admission, switching VTE prophylaxis to therapeutic anticoagulation following local protocol, and ICU admission due to ARDS did not differ among patients with positive aPLs compared to those with negative aPLs (18% vs. 16%, adjusted odds ratio 0.98, 95% CI 0.1–6.7; *p *value = 0.986), as shown in Table 3. Likewise, the presence of aPLs did not affect the secondary outcomes, including all-cause mortality, venous thrombosis, arterial thrombosis, ICU discharge, time to mortality, and time to ICU discharge, as demonstrated in Table 3.

**Table 3:** Clinical outcomes of critically ill COVID-19 patients (*n* = 60)

Primary outcome during ICU stay	aPL(s) positive (*n* = 22), *n* (%)	aPL(s) negative (*n* = 38), *n* (%)	Crude OR, (95% CI)	*p* value	Adjusted OR^a^	*p* value
A composite of mortality or thrombosis	4 (18)	6 (16)	1.2 (0.3–4.8)	0.811	0.98 (0.1–6.7)	0.986

Secondary outcomes during ICU stay	aPL(s) positive (*n* = 22),* n* (%)	aPL(s) negative (*n* = 38),* n* (%)	*p* value
All-cause mortality	2 (9)	5 (13)	1^*^
Venous thrombosis	1 (5)	1 (3)	1^*^
Arterial thrombosis	2 (9)	1 (3)	0.584^*^
ICU discharge	19 (86)	32 (84)	1^*^
Time to mortality—mean ± SD [days]	22 ± 9	27 ± 17	0.711
Time to ICU discharge—median (IQR) [days]	8 (6)	8 (9)	0.945

*OR* odds ratio, *aPL* antiphospholipid antibody, *ICU* intensive care unit

^a^Adjusted for the use of venous thromboembolism (VTE) prophylaxis prior to ICU admission, switching VTE prophylaxis to therapeutic anticoagulation following local protocol, and admission to ICU due to acute respiratory distress syndrome; ^*^p value calculated using Fisher’s exact test

#### Prevalence of antiphospholipid antibodies among critically ill patients with COVID-19 during the development of thrombosis

The repeated screening of aPLs during the development of thrombosis revealed that among two patients who developed venous thrombosis, anticardiolipin IgG/IgM and lupus anticoagulant were detected in one patient, and among three patients who developed arterial thrombosis, only lupus anticoagulant was detected in one patient, whereas the remaining antibody screening was negative for the remaining patients, as demonstrated in Table 4.

**Table 4:** Prevalence of antiphospholipid antibodies among critically ill COVID-19 patients at the time of thrombosis (*n* = 5)

aPL(s)	Arterial thrombosis (*n* = 3), *n* (%)	Venous thrombosis (*n* = 2), *n* (%)
IgG anti-β2GPI	0	1 (50)
IgM anti-β2GPI	0	1 (50)
IgG aCL	0	0
IgM aCL	0	0
Lupus anticoagulant	1 (33)	1 (50)

*aPL* antiphosphoilipid antibody, *anti-β2GPI* anti-b2 glycoprotein-I antibody, *aCL* anticardiolipin antibody

## Discussion

Since the beginning of the COVID-19 pandemic, arterial and venous thrombosis associated with COVID-19 infection due to excessive inflammation, hypoxia, and diffuse DIC have been proposed [2, 14, 19, 20]. One of the early reported coagulation derangement is the presence of aPLs [7]. Nevertheless, the prevalence and the clinical significance of aPLs among patients with COVID-19 infection have not been well established yet [2, 8–12, 14, 20, 21]. To this end, we attempted to investigate this subject in critically ill patients with COVID-19. In this prospective observational study that included critically ill patients with COVID-19, the prevalence of aPLs was found to be 37%, with lupus anticoagulant being the most commonly detected aPL, without increasing the risk of mortality or thrombosis compared to critically ill patients with negative aPLs.

The prevalence of aPLs in critically ill patients with COVID-19 is a matter of debate in the literature [7–11, 14, 21]. According to our data, we detected aPLs in 37% of the study population, with lupus anticoagulant identified in 35% of the study population. Bowles L et al*.* in May 2020 in the United Kingdom reported that the lupus anticoagulant was detected in 91% of patients with COVID-19 with prolonged aPTT [9]. Conversely, Xiao M et al*.* recently reported that aPLs were detected in 47.0% among critically ill patients, and lupus anticoagulant was detected in only 3% of critically ill patients [10]. Furthermore, the reported subtypes of aPLs also greatly varied in the literature [9, 10]. In our study, lupus anticoagulant was the most commonly identified among the screened antibodies as it was detected in 21 (35%) of the study population, whereas anti-β2-glycoprotein IgG/IgM was detected in one patient only, and no anticardiolipin antibodies were detected. Similar to our findings, Devreese et al*.* recently found single lupus anticoagulant positivity in around 52% of critically ill patients with COVID-19 [11]. In contrast, Xiao M et al*.* found that in critically ill Chinese patients with COVID-19, lupus anticoagulant and anticardiolipin IgA were detected in 3% and 25.8% of patients, respectively [10]. Additionally, anti-β2-glycoprotein IgA was the most prevalent antibody, with an incidence of 28.8% of their study population [10]. In fact, anti-β2-glycoprotein IgA is commonly identified in particular ethnic groups [7, 22]. Specifically, in a large Chinese cohort, anti-β2-glycoprotein IgA was identified in 39% of patients with APS [23]. Nevertheless, anti-β2-glycoprotein IgA and anticardiolipin IgA are not part of the laboratory criteria for the APS diagnosis as IgA aPLs are not clearly associated with clinical manifestations of APS [16]. Consequently, we have not screened our study population for IgA aPLs.

We found that the presence of aPLs did not increase thrombosis among critically ill patients with COVID-19, with an overall low incidence rate of arterial and venous thrombosis (8%, 5/60). In comparison to the incidence of thrombosis among critically ill patients with COVID-19 in the Netherlands, the incidence rate of thrombosis reported in our study is considered low [24]. Likewise, Helms J et al*.* reported an incidence rate of 43% of clinically relevant thrombotic complications among patients with COVID-19 admitted to ICU [25]. This could be attributed to the approach of detecting thrombosis in COVID-19 patients in our study, as we did not utilize a systematic approach. A few COVID-19 studies have evaluated the use of a systemic screening protocol to detect thrombosis using imaging studies for all the study population regardless of symptoms that detected missed thrombotic events [26–28]. In addition, the local protocol of VTE prophylaxis followed by COVID-19 facilities in Qatar permits the escalation to therapeutic doses of anticoagulation without a confirmed thrombosis diagnosis, which could have contributed to the lower reported thrombotic rate in our study. Nevertheless, we adjusted for this variable in the primary analysis to assess the influence of aPLs on thrombosis.

Upon ICU admission, we found that fibrinogen and CRP levels were significantly different between the two groups, with higher values in the antiphospholipid-positive group. Similar to our findings, Miesbach et al*.* reported elevated CRP levels in the presence of aPLs [29]. Nevertheless, the high levels of CRP in the antiphospholipid-positive group could have interfered with the detection of the lupus anticoagulant in the study population, resulting in false positive detection of lupus anticoagulant in acute phases [30, 31]. Ames et al*.* concluded that high fibrinogen levels are detected in antiphospholipid-positive patients with thrombosis [32]. In spite of that, this was not associated with an increased risk of thrombosis or mortality in the antiphospholipid-positive group, making the clinical significance of such observation doubtful.

In our study population, 95% of antiphospholipid-negative patients were on VTE prophylaxis before ICU admission compared to 48% in antiphospholipid-positive patients. This significant difference is attributed to the location of the patient before ICU admission; majority of patients were transferred from the medical ward to ICU were in the antiphospholipid-negative group (80%) where VTE prophylaxis was initiated. The rest were admitted from home or non-medical quarantine facilities directly to the ICU, and they constitute a larger proportion of the antiphospholipid-positive group. Despite its statistically significant difference, VTE prophylaxis before the ICU admission did not affect the primary outcome as mortality and thrombosis events remained unaffected after adjustment for this variable.

This study has a few limitations. First, the sample size calculation was not based on local data on the prevalence of aPLs in Qatar, and this proposed a limitation as the reported prevalence in our study was less than that used for study power calculation, which may have led to a lower study power than anticipated. Second, aPL screening was not repeated at 12 weeks to confirm the persistence of these antibodies due to difficulty in following-up the patients post discharge in view of strict infection control measures implemented in the country, especially that all regular medical follow-up visits became virtual through telemedicine during the COVID-19 outbreak. Third, the follow-up period was set to 60 days, mortality, or until ICU discharge, whichever came first. A longer duration that extends beyond the ICU discharge might have allowed the detection of more thrombotic events, if any. Fourth, the evaluation of lupus anticoagulant was performed during an acute phase, which could have interfered with the detection of lupus anticoagulant. Finally, we reported a low thrombotic rate which might have resulted from not using a systematic approach to detect thrombosis. Nevertheless, our study answered a crucial question for clinicians to comprehend the role of aPL antibodies in COVID-19 induced thrombosis, and found that the presence of aPL antibodies did not increase the risk of thrombosis or mortality in critically ill patients with COVID-19, which can serve as a useful guide for rheumatologists, in particular, as our results have ascertained that the presence of aPL antibodies during COVID-19 is not involved in the hemostatic abnormalities in COVID-19.

## Conclusion

aPLs do not seem to affect the outcomes of patients with COVID-19 in the ICU in terms of all-cause mortality and thrombotic events, whether arterial or venous thrombosis. Therefore, clinicians, especially rheumatologists who are involved in confirming the diagnosis of APS, may defer routine screening for APS among patients with COVID-19 unless deemed clinically appropriate. Studies with longer follow-up and repeated aPL screening at 12 weeks are needed to confirm the study’s findings.

## Data Availability

All data analyzed during this study are included in this published article. Individual participant data will not be publicly available due to ethical restrictions by HMC medical research center that does not generally allow sharing of data to individuals or entities outside the HMC.
